# Cognitive Bias Modification for paranoia (CBM-pa): study protocol for a randomised controlled trial

**DOI:** 10.1186/s13063-017-2037-x

**Published:** 2017-06-29

**Authors:** Jenny Yiend, Antonella Trotta, Christopher Meek, Ilvana Dzafic, Nora Baldus, Bryony Crane, Thomas Kabir, Daniel Stahl, Margaret Heslin, Sukhwinder Shergill, Philip McGuire, Emmanuelle Peters

**Affiliations:** 1 Department of Psychosis Studies, Institute of Psychiatry, Psychology and Neuroscience, King’s College London, London, UK; 2 Queensland Brain Institute, University of Queensland, Brisbane, QLD Australia; 3 The McPin Foundation, London, UK; 4 Health Service and Population Research Department, Institute of Psychiatry, Psychology and Neuroscience, King’s College London, London, UK; 5 Department of Psychology, Institute of Psychiatry, Psychology and Neuroscience, King’s College London, London, UK

**Keywords:** Cognitive Bias Modification, Paranoia, Psychosis, Computerised therapy, RCT, Interpretation bias, Persecutory delusions

## Abstract

**Background:**

Persecutory delusions are the most common type of delusions in psychosis and present in around 10–15% of the general population. Persecutory delusions are thought to be sustained by biased cognitive and emotional processes. Recent advances favour targeted interventions, focussing on specific symptoms or mechanisms. Our aim is to test the clinical feasibility of a novel psychological intervention, which manipulates biased interpretations toward more adaptive processing, in order to reduce paranoia in patients.

**Methods:**

The ‘Cognitive Bias Modification for paranoia’ (CBM-pa) study is a feasibility, double-blind, randomised controlled trial (RCT) for 60 stabilised outpatients with persistent, distressing paranoid symptoms. Patients will be randomised at a 50:50 ratio, to computerised CBM-pa or a text-reading control intervention, receiving one 40-min session per week, for 6 weeks. CBM-pa involves participants reading stories on a computer screen, completing missing words and answering questions about each story in a way that encourages more helpful beliefs about themselves and others. Treatment as Usual will continue for patients in both groups. Patients will be assessed by a researcher blind to allocation, at baseline, each interim session, post treatment and 1- and 3-month follow-up post treatment. The primary outcome is the feasibility parameters (trial design, recruitment rate and acceptability) of the intervention. The secondary outcomes are clinical symptoms (including severity of paranoia) as assessed by a clinical psychologist, and ‘on-line’ measurement of interpretation bias and stress/distress. The trial is funded by the NHS National Institute for Health Research.

**Discussion:**

This pilot study will test whether CBM-pa has the potential to be a cost-effective, accessible and flexible treatment. If the trial proves feasible and demonstrates preliminary evidence of efficacy, a fully powered RCT will be warranted.

**Trial registration:**

Current Controlled Trials ISRCTN: 90749868. Retrospectively registered on 12 May 2016.

**Electronic supplementary material:**

The online version of this article (doi:10.1186/s13063-017-2037-x) contains supplementary material, which is available to authorized users.

## Background

Psychosis is one of the most disabling mental health conditions, with a lifetime rate of 3.5% [1], and it is associated with significant distress, unemployment, impaired social functioning, physical ill health and suicidal ideation [2]. Persecutory delusions are the most frequent and clinically significant symptoms of psychosis. They are associated with considerably more distress than other types of delusion [3]; are most likely to be acted upon [4] and represent a strong predictor of hospitalisation [5]. Over one third of all UK psychiatric patients suffer from persecutory delusions, and they are reported in depression [6], bipolar disorder [7], posttraumatic stress disorder [8], anxiety [9], and with the highest prevalence and greatest intensity in schizophrenia [10]. A significant proportion of patients suffering from persecutory delusions, continue to experience distressing symptoms despite available treatments [11, 12].

Previous research showed that the distribution of paranoid thinking in the general population is continuous, ranging from social evaluative concerns (e.g. fears of rejection) to severe threat (e.g. people trying to cause significant harm), indicating a hierarchical structure to paranoia [13–17]. Persecutory delusions are the extreme point on the continuum of paranoid belief, and are likely to be perpetuated by biased cognitive-emotional processes [13–18].

‘Cognitive bias’ refers to selective processing of information matching the core content of the pathology of a disorder; for example, individuals with threat-related persecutory delusions interpret emotionally ambiguous information as threatening. Savulich et al. [17] point out that ‘cognitive deficits are impairments in cognitive functioning at the global level, whereas cognitive biases involve the selective processing of pathology-congruent information with the potential to confirm matching pathological beliefs’ (p. 516).

For example, general problems in attentional functioning or working memory are cognitive deficits, but repeatedly attending to a certain type of maladaptive stimulus, in preference to more benign alternatives, or consistently interpreting emotionally ambiguous information in one (maladaptive) direction would both be examples of a cognitive bias (in attention and interpretation, respectively). Furthermore, recent theoretical thinking in psychosis is attempting to integrate cognitive models of threat appraisals with models based on neural findings in areas such as the amygdala, the insula, the hippocampus, the anterior cingulate cortex and the prefrontal cortex [19].

These cognitive biases help to cause and maintain a range of psychopathologies [20, 21], including psychosis/delusions [3]. Cognitive therapy can work by changing underlying biases so that these maintaining mechanisms are no longer present and, ideally, patients instead acquire biases that promote wellbeing [22, 23] and, as a result, symptoms resolve. However, the Schizophrenia Commission report that the NICE-recommended psychological therapy for psychosis, cognitive behavioural therapy (CBT), is received by only 1 in 10 of those who could benefit and has shown only moderate effect sizes for delusions [24, 25]. New directions in treatments for delusions emphasise briefer, targeted interventions, with a focus on putative causal factors, such as cognitive biases [26, 27].

Here, we aim to test the feasibility of a new targeted psychological intervention ‘Cognitive Bias Modification for paranoia’ (CBM-pa) which uses a computerised task to manipulate biased interpretations of emotionally ambiguous information, with the aim of promoting more adaptive processing. CBM is a validated, theory-driven treatment with an established methodology [28]. CBM has arisen directly from laboratory research into the nature of the cognitive biases across a range of disorders. Two high-impact articles have alerted the research community to CBM’s potential as a novel treatment [29, 30], with evidence of its efficacy in reducing pathological beliefs, symptoms and stress vulnerability in anxiety and depression [31]. More cautionary reports have also pointed out that efficacy varies across studies [32] and researchers need to proceed with caution in applying CBM to new clinical populations, ensuring that the necessary basic research has been conducted prior to implementing CBM methodologies.

The first necessary step in this basic research is to identify the relevant naturally occurring biases and how they present in the target disorder. The CBM methodology can then be adapted to be specifically relevant for that target disorder. Previous studies suggest that a negative interpretation bias occurs in those with elevated vulnerability to paranoia, and that this bias may be strongest for material matching paranoid beliefs both in nonclinical and clinical samples [17, 18]. Specifically, interpretation of everyday information as personally threatening has been found to be related to the onset and maintenance of paranoid symptoms [33]. Therefore, an intervention targeting these biases offers potential to reduce distressing paranoia. Moritz and colleagues [34], have pioneered ‘metacognitive training’ (MCT) for a range of social-cognitive biases found in psychosis. Using group training, they obtained improvements in delusions in a large randomised controlled trial (RCT). Previous studies, using computerised treatment targeting reasoning bias in patients with persecutory delusions, also demonstrated the effectiveness of a brief reasoning intervention in improving both reasoning processes and paranoia [35, 36].

However, the use of CBM to modify interpretations of material specifically relevant to paranoia and paranoid beliefs remains unexplored and CBM-pa was designed to fill this gap.

### Feasibility, double-blind randomised controlled trial

The current study is a feasibility, double-blind RCT of CBM-pa with patients who suffer from persistent, distressing paranoid symptoms. The CBM-pa intervention is delivered on a computer, set up by the researchers and, thereafter, is a self-directed, automated package, self-administered by the patient. The intervention uses ambiguous scenarios which could potentially be interpreted in a manner that reinforces paranoia, or instead could be interpreted in more benign way. While patients read these scenarios they are asked to complete experimental tasks (word completion and comprehension questions) which implicitly influence the interpretation that is actually made. Additional file 1: Figures S1a–h shows an example trial from the intervention. The intervention aims to reduce paranoid beliefs, paranoid symptoms and stress/distress, and will be evaluated in comparison to a text-reading control (see Additional file 1: Figure S1i). Both conditions will be conducted in addition to Treatment as Usual (TAU) which will be in the form of individualised combinations of medication and care coordination. This design directly evaluates the hypothesised ‘active ingredient’ (bias reduction) by using the control condition to match all other aspects of the adjunct intervention.

The main objective of this feasibility study is to test the integrity of the protocol in terms of length and feasibility. A secondary objective is a preliminary evaluation of efficacy. For this the primary outcome is the severity of paranoid symptoms. A third objective is the selection of primary outcomes (e.g. paranoid symptoms, interpretation bias and vulnerability to stress) for the subsequent trial based on consensus after presentation of results to the Steering Group. This decision will be informed by (1) observed effect sizes and (2) opinions on the most important outcome from qualitative interviews. The necessary withholding of information about group assignment might be a significant issue because heightened suspiciousness is an inherent feature of clinical paranoia.

Estimates of population variances of the main outcomes for future power calculations will be based on Browne [37], who suggests using the upper 80th percentile of confidence intervals around the estimates.

## Methods

The study has been approved by the London – City Road and Hampstead Research Ethics Committee on 26 February 2016 (reference: 16/LO/0071) and has been registered (Current Controlled Trials, ID: ISRCTN90749868). Amendments will be submitted to both the Ethics Committee and the Clinical Trials Register. Informed consent will be obtained from all participants. A Trial Steering Group (TSG) and LEAP (Lived Experience Advisory Panel) have been formed. The TSG also functions as a Data Monitoring Committee by assessing, at regular intervals, the progress of the study, participant safety, feedback and complaints, study milestones and, if relevant issues arise, whether to continue, modify, or stop the trial.

### Participants and study setting

The primary endpoints of this feasibility trial are factors that affect successful trial conduct, rather than measures of intervention effects. Hence, power analyses for intervention outcomes were not undertaken in advance. The study recruitment target is 60 participants, consistent with the widely accepted sample size of 20–40 participants for pilot studies [38, 39], allowing for some dropout. Assuming a follow-up rate of 70% or more, a one-sided 95% confidence interval for this key feasibility parameter will extend no more than 10% from the observed proportion. A minimum sample size of 30 is sufficient to obtain robust estimates of population variances for future power calculations [37, 38]. Therefore, recruiting 60 patients in this pilot trial should be sufficient to provide estimates of population variance on key outcomes.

Participants will be recruited from a range of local sources, including: Institute of Psychiatry, Psychology and Neuroscience (IoPPN) and South London and Maudsley (SLaM) research registers; SLaM NHS Foundation Trust services; service users’ networks and voluntary sector organisations. Patients will be screened and selected for invitation to participate by researchers according to the inclusion and exclusion criteria. The inclusion criteria are:

1. Any diagnosis featuring clinically significant persecutory or paranoid symptoms, present for at least the preceding month. In order to be included in the study, participants had to score 3 or more on the Positive And Negative Syndrome Scale (*PANSS*) item 6 [40] of the positive symptoms scale and which measures ‘suspiciousness/persecution’. Paranoid symptoms were assessed over the telephone by a trained researcher. A score of 3 reflects ‘mild’ paranoid symptoms as expressed by ‘a guarded or even openly distrustful attitude’;
2. Displaying a baseline interpretation bias. A link is sent to potential participants to complete a bias screening task online (or, for those without Internet access, participants are asked to come to the IoPPN and complete it using local laptops). The bias screening task is a shortened (eight-item) version of the *Similarity Rating Task* (*SRT*) [41] delivered using the on-line data collection software Qualtrics. Shortened versions have demonstrated adequate reliability in previous work [42]. The items used in this task were sourced by randomly selecting from the original CBM-pa training items used in sessions 1, 2 or 3. It was ensured that the items chosen were distinctive, and had an even number of yes/no correct responses. In the screening *SRT*, sentences reflecting non-paranoid (i.e. benign) and paranoid interpretations were denoted T+ and T−, respectively, and rated by participants on a 1–4 Likert scale (higher score = stronger endorsement). An interpretation bias screening score is calculated: T+ minus T−. Screening bias scores, therefore, range from +3 to −3 with positive and negative scores reflecting bias favouring nonparanoid and paranoid meanings, respectively. A bias score of 1 or below during the *SRT* at screening is necessary to be selected for the study. This cutoff has been adopted in order to exclude anyone showing a strong positive interpretation bias. The screening version of the *SRT* differed slightly from the baseline version, in that there were only eight *SRT* items and no foils
3. Stable on all prescribed medication (including anti-psychotic) for at least the last 3 months and expected to be so for study duration
4. Age range of 18–65 years
5. Capacity to consent defined as the ability to use and understand information to make a decision, and communicate any decision made. This is assessed by a trained research assistant (CM) and a clinical psychologist (AT) according to the Mental Capacity Act [43]

Exclusion criteria are:

1. Severe cognitive impairment, or illiteracy
2. Major physical illness (cancer, heart disease, stroke)
3. Major substance or alcohol misuse, and
4. Currently receiving, or soon due to receive, a psychological therapy targeting the same psychological mechanisms as CBM-pa (paranoid beliefs), or having done so in the last 3 months

Psychological therapy is defined as five or more consecutive sessions with a trained therapist, either in an individual or group setting, for the purpose of alleviating symptoms arising from paranoia or related issues. Receipt of therapy for any other purpose (e.g. occupational or art therapy; counselling) will be assessed on a case-by-case basis, against the principle that eligibility requires any cognitive mechanisms targeted by therapy to be independent of those targeted by CBM-pa.

### Randomisation and blinding

We will conduct this study following Consolidated Standards of Reporting Trials (CONSORT) guidelines [44]. The King’s Clinical Trials Unit will conduct double-blind randomisation. CBM-pa is self-administered, allowing researchers to be blind to condition. Control and intervention are designed to appear procedurally identical. Blinding will be implemented by the creation of 60 unique, randomly labelled computer programmes which mask the assigned treatment arm from researchers. Should occasional instances of unblinding accidentally occur, these can be reported without jeopardising the status of the remainder of the sample. Participants and researchers will be asked to guess the assigned condition to evaluate the success of double-blinding at the end of all data collection. Adaptive randomisation by minimisation will be used that allocates subjects to the treatment group that best maintains balance in stratifying factors, such as: gender, severity of baseline paranoia measured by the *PANSS* item 6 [40], severity of interpretation bias measured by the *SRT* [41].

## Interventions

**Fig. 1:**
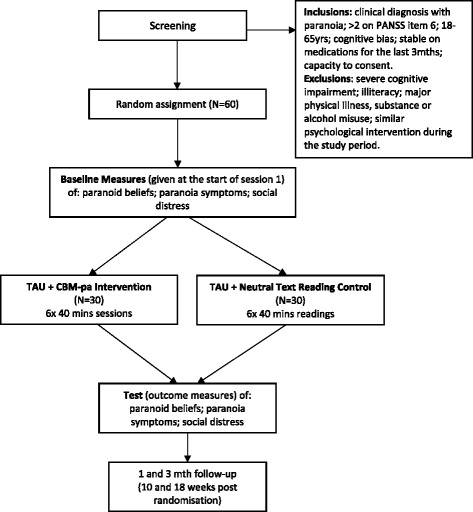
Trial flow diagram

Participants will attend the Institute of Psychiatry, Psychology and Neuroscience, King’s College London, or the NIHR/Wellcome Trust King’s Clinical Research Facility, for six sessions and receive postal/telephone 1-and 3-month follow-ups (respectively at 4 and 12 weeks from end of last session). See Fig. 1 for the trial flow diagram.

Groups should be equalised on the expectation of benefit because the introduction to each trial arm is delivered by computer and standardised across the two conditions. Expectation of benefit will be assessed using the *Credibility and Expectancy Questionnaire* [45]. The intervention will be delivered on laboratory-owned laptops.

Qualitative interviews (*n* = 8) will be conducted after the end of quantitative data collection, providing an in-depth assessment of participants experience of the intervention, including potential side effects (e.g. unexpected emergence, rather than amelioration of paranoid thoughts).

### Treatment-as-Usual (TAU) + CBM-pa

We will record details of TAU received during the study period using a standardised template to record participants’ responses to a series of set questions about treatment received, including pharmacotherapy and any treatment changes. The first 10 participants consenting for access to notes will be used to assess feasibility of case-note data collection to record TAU.

#### CBM-pa procedure and item development

CBM-pa (see Additional file 1: Figures S1a-h) consists of 40 unique training items completed per session (duration 40 min plus midpoint break), for six weekly sessions, involving a total of 240 different items.

To maximise clinical relevance, the LEAP group developed over 100 examples of everyday situations drawn from their real experiences to be used as content for the six study intervention sessions. Training items were selected from these and further developed by the research team into the format required for use as a training item (see example given in Additional file 1). The items were arranged into six topic areas/categories of paranoia, namely:

1. Physical harm (37 items): e.g. being poisoned, injured, threatened by terrorism, killed, etc.
2. Social/interpersonal threat (87 items): e.g. being gossiped about, judged, disliked, dumped, fired
3. Medical/paramedical/health care threat (19 items): e.g. being prescribed drugs with adverse effects, being conspired against by medical staff conspire against you, having one’s deoxyribonucleic acid (DNA) stolen
4. Threat of persecution/spying (40 items): e.g. being stalked, spied on, followed, arrested by police
5. Delusions of reference/magical thinking (15 items): e.g. interpreting stimuli as personal messages
6. General suspiciousness/distrust not relating to physical threat or persecution (42 items): e.g. being robbed, plagiarised, irritated by neighbours

Items were independently rated, according to their severity, by two clinical psychologists (EP, AT). The intra-class correlation coefficient for the severity ratings (ICC) was 0.992 (indicative of strong inter-rater reliability). Training items were distributed across sessions in terms of their mean severity ratings such that items become more severe from one session to the next, based on previous research findings suggesting this is more acceptable to participants with pre-existing negative biases [42].

A typical item is shown in Additional file 1: Figure S1b. Each item depicts an emotionally ambiguous situation, is interactive and involves participants reading three lines of text on a computer screen, then completing a missing word and answering a related question (yes/no). Both the word completion and the question encourage participants to interpret the ambiguity inherent in the situation in a manner that promotes helpful beliefs about themselves and others (see example given in Additional file 1: Figures S1a − h). Equal numbers of ‘yes’ and ‘no’ question responses are presented randomly to avoid participants developing a fixed response set. Word completions were unique (no repetitions) and constructed by removing all vowels, then reinstating one in cases where LEAP feedback indicated that the resulting difficulty level was too high. In response to further feedback from LEAP a ‘clue’ was created for each item by adding more letters to encourage the participant to try again following an incorrect response.

In order to reflect the increasing drill down towards core beliefs that is used in traditional cognitive therapies, sessions 1 and 2 are delivered using the word ‘thoughts’ (e.g. ‘You fly to a business meeting abroad. You plan to attend the guest reception but arrive 20 min late. Upon entering the reception you look around and *think* everyone looks…’); 3 and 4 using ‘assumptions’ (e.g. ‘You are running late for an appointment and rush to get to the bus stop on time. As you approach, gesturing for the bus to wait, you see the driver look in the mirror and pull away, smiling. You *assume* that the bus driver just…’) and 5 and 6 using ‘beliefs’ (e.g. ‘You are sitting at a bus stop when a group of teenagers walk over and light up their cigarettes. As you cough loudly, they throw the empty carton saying ‘smoking kills’ towards you and laugh. You *believe* that this is…’).

### Treatment-as-usual (TAU) + text-reading control

A computerised text-reading control will be given to those randomised to this condition (see Additional file 1: Figure S1i). The experience is identical to CBM-pa, but content omits the active ingredient: resolution of an emotionally ambiguous situation in a benign/non-paranoid manner. Instead control participants read and respond to factual material. Similarly to CBM-pa, training items were divided into six sessions of 40 items each. It was ensured that there are no obvious systematic differences between the control and intervention items (equal number of yes/no answers in each session, no word repetitions in the incomplete words, each item is three sentences long over three lines, topics are evenly distributed between each session).

The LEAP group reviewed all items to be used in the study intervention and control arm for readability and suitability and tested the software to be used in the study for acceptability.

## Outcome measures and timeline

**Fig. 2:**
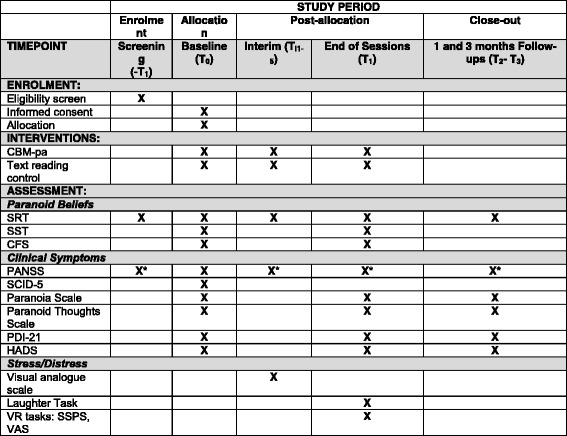
Standard Protocol Items: Recommendations for Interventional Trials (SPIRIT) diagram

The Standard Protocol Items: Recommendations for Interventional Trials (SPIRIT) diagram provides an overview of the measures used in the trial and their time points (see Fig. 2). To evaluate the optimum number of sessions, dose-response effects will be assessed by measuring paranoid symptoms and interpretation bias at each session (i.e. what ‘dose’ of interpretation bias training achieves the best reduction in paranoia) using the secondary outcome measures (see below). This will inform decisions about number of sessions for full trial.

### Primary outcomes

As the study is a feasibility evaluation of the CBM-pa intervention there is no single primary outcome measure. Instead, feasibility will be assessed on the basis of rates of recruitment, consent, dropout and follow-up. In addition, acceptability of randomisation will be examined, including the integrity of double-blinding. Qualitative interviews will be conducted after the end of quantitative data collection, using a purposively selected subsample of eight active-arm patients. Purposive selection will represent sample variation in the following priority order: gender, severity of paranoia, severity of bias, age and ethnicity. The aim is to explore acceptability, barriers to participation, promoting engagement and optimum form of delivery of the intervention.

### Secondary outcomes

The study will sample three domains of possible outcomes: interpretation bias, clinical symptoms and stress/distress. For each we will collect information on (1) comprehensibility and suitability and (2) variance estimates by calculating standardised treatment effect sizes (Cohen’s *d*) and their confidence intervals.

Interpretation bias will be measured using the:

- *Similarity Rating Task (SRT)* [41]. This is a standard test used to measure interpretation bias. Items describe ambiguous social scenarios that permit either paranoid- or non-paranoid interpretations and were developed by Savulich et al. [17], based on Freeman and Garety’s [46] definition of paranoia as a person’s belief that intentional ‘harm is occurring, or is going to occur’. Items are shown to have sensitivity to detect differences between high-paranoid and low-paranoid groups of participants who were found to differ in their interpretation bias on paranoid item content [17], such that the high-paranoid group showed a significantly more maladaptive pattern of interpretation (reduced endorsement of positive and non-paranoid interpretations) compared with the low-paranoid group

For the ‘encoding’ part of the task participants read 12 ambiguous passages, complete one of the last word’s missing letters and answer a comprehension question. An example of such a passage is entitled ‘car park’, followed by ‘You have parked your car in the supermarket car park and just done your weekly food shopping. On your return to your car you notice a man sat in a nearby car. He is sitting alone and looking out the w-nd-w.’ Hence, for each item, the third sentence ends with a word that has several letters missing. Participants are asked to indicate the first missing letter. For the ‘Car park’ example, the letter ‘i’ would have to be indicated to show that the last word was ‘window’. In order to check that participants correctly encoded the passage, they had to choose ‘yes’ or ‘no’ as an answer to a comprehension question, such as ‘Have you just been to the supermarket?’ which would be correctly answered with ‘yes’ in the ‘Car park’ example. Participants are given feedback on the screen about the correctness of their response to this comprehension question.

For the ‘recognition’ part of the task, participants are given four sentences, two of which represented ‘targets’ that capture a paranoid (T−) and a non-paranoid (T+) interpretation of the original passage, respectively. The other two sentences represent ‘foils’ that contain either paranoid- or non-paranoid content that is unrelated to the corresponding passage. These foils are used to control for response bias which refers to the tendency to, for instance, endorse information that is congruent with a paranoid schema rather than actually interpreting the specific item in a paranoid way [17]. For the ‘Car park’ example, the corresponding target paranoid interpretation is ‘The passenger does not want to sit next to you’ (T−) and the corresponding non-paranoid interpretation is ‘The passenger wants more legroom’ (T+). ‘Your friend is ignoring you’ and ‘Your friend is busy at work’ represent a paranoid foil (F−) and a non-paranoid foil (F+), respectively. Participants are asked to rate ‘how similar in meaning from 1 = very different in meaning to 4 = very similar’, each of the four sentences is to the corresponding original passage. By comparing participants’ ratings of each of the four sentences, we can infer their spontaneous interpretation of the original passage. Interpretation bias scores can be calculated as described above (T+ minus T−) for the screening version of this task.

- *Scrambled sentences task (SST)* [47, 48]*.* In this pen-and-paper task participants are presented with ‘nonsense’ sentences, each comprising six words and are asked to select and re-order five of these words to create a grammatically correct sentence. For example, ‘pleasant to people hostile me are’ could become ‘people are hostile to me’ (paranoid interpretation) or ‘people are pleasant to me’ (non-paranoid interpretation). There are fifteen sentences in total and participants have 5 min to complete as many as possible. The order of words selected is indicated by participants writing the numbers ‘1’ to ‘5’ above the words. Instructions to participants ask them to ‘choose whatever comes to mind first’ and form statements rather than questions. Before unscrambling the 15 sentences, participants are asked to memorise a six-digit number (e.g. 615239) which they are told to keep in mind while doing the task and which they are asked to recall after completion of the task. Rude et al. [47] showed that the addition of this cognitive load makes the task more sensitive to detecting interpretation biases possibly because it stops participants suppressing biases. For instance, negative interpretation bias on the *SST* only predicted depression if the *SST* was performed under cognitive load [47]

Two values are recorded: (a) the total number of grammatically correct sentences created with a paranoid meaning is recorded for each participant (including those using fewer than five words, such as ‘people are hostile’) and (b) the total number of items attempted within the available time. From these values a negative (paranoid) interpretation bias score is calculated as a proportion: [A/B] × 100. Bias scores, therefore, range from 0 to 100, with higher values reflecting bias favouring paranoid interpretations.

- *Cognitive Flexibility Scale (CFS)* [49]. This self-report measure consists of 12 items that participants rate on a 6-point scale ranging from 1 (strongly disagree) to 6 (strongly agree). It measures the three components of cognitive flexibility: (a) awareness that in any given situation there are options and alternatives available, (b) willingness to be flexible and adapt to the situation and (c) self-efficacy in being flexible

Psychotic symptoms are measured at baseline using the *PANSS* [40] conducted by a trained clinical psychologist. This is a 30-item clinical tool that measures symptom severity in psychosis. It consists of three subscales with seven items for positive symptoms, seven items for negative symptoms and 16 items for general symptoms. Each symptom is rated on a 7- point Likert scale from 1 (symptom absent) to 7 (symptom extreme), with a minimum score of 7 and a maximum score of 40 for the *PANSS* subscales; and a minimum score of 16 and a maximum score of 112 for the general symptoms.

Specifically, severity of paranoid symptoms is assessed by *PANSS* item 6 (P6 Suspiciousness/Persecution) which can be used in isolation to evaluate degree of clinical paranoia. This item measures unrealistic or exaggerated ideas of persecution using a 7-point Likert scale where 1 = absent; 2 = minimal, may be at upper extreme of normal limits; 3 = mildly symptomatic, then incrementally upwards to 7 = extreme persecutory delusions.

In addition to the *PANSS* item 6, the following self-report scales will be used to assess paranoid symptoms: the *Paranoia Scale* [50], the *Paranoid Thoughts Scale* [51] and the *Peters Delusions Inventory* (*PDI-21*) [52]. Although the latter is not a clinical symptom measure, it includes subscales reflecting clinically important elements of delusional ideation: degree of delusional conviction, frequency of delusional thoughts and degree of associated distress.

Symptoms of depression/anxiety are assessed using the *Hospital Anxiety and Depression Scale (HADS)* [53].

Stress/distress is assessed using three measures:

- *Virtual Reality Environment* (*VRE*). Participants wear a headset through which they view a three-dimensional social scene populated by neutral characters (a cafe). Participants spend approximately 4 min in the virtual environment and can move through the virtual environment by walking and whole body turning. *Visual Analogue Scale* (*VAS*) measures of state mood assess how anxious, sad, paranoid and friendly participants feel and are given immediately before and after experiencing the virtual environment. Participants also complete the *State Social Paranoia Scale* (*SSPS*) [54]. The 10 items for this measure of recent paranoid thinking contain both elements of threat and intention (e.g. ‘Someone had it in for me’, ‘Someone stared at me in order to upset me’, ‘Someone was trying to isolate me’, ‘Someone was trying to make me distressed’). Each item is scored on a 5-point scale (Do not agree – Totally agree). Higher scores indicate greater levels of persecutory thinking [13, 55]
- *Laughter task* [56]. Two experimental events are presented during the testing session. These are (a) an interruption by a female stooge calling the experimenter out of the room and (b) following the exit of the stooge and the experimenter, a 35-s audio recording of male and female laughter played outside the room. The recording is played using an iPad and is identical for each participant. The laughter was piloted in a sample of five individuals (without a history of mental illness and with English as a first language) to ensure that it was audible to those inside the room without sounding so loud so as to seem artificial. The aim of the task is to expose participants to two ‘real-life’ events that are inherently ambiguous by nature. At the end of the testing session participants are asked a series of questions to assess their explanations for these two events. Verbatim explanations given by individuals for each of the events are rated for the presence of a paranoid attribution (an idea of reference or an idea of persecution)
- *Visual Analogue Scales*. Anxious, sad, paranoid and friendly state mood will be assessed using a simple *VAS* pre and post each training session to determine the immediate subjective impact on participants of taking part in the intervention. Furthermore, the academic literature suggests that state emotion may influence paranoia-relevant biases [57] and intervention effects might, therefore, be moderated by emotional experience during training

#### Procedures

The assessment procedure is outlined in Fig. 2. The first contact with participants is during screening, where we check for participants’ eligibility by assessing the inclusion and exclusion criteria described above. Based on this screening contact, researchers complete a capacity assessment to confirm that they perceived participants to understand and retain information sufficiently to make an informed choice about taking part in the study. Following informed consent, baseline assessment (T_0_) will be completed prior to randomisation, at the start of session 1, and will include measures of:

- Sociodemographic information such as age, gender, ethnicity, IQ [58], educational level, employment and relational status
- Paranoid beliefs (*SRT*, *SST* and *CFS*). Twelve *SRT* items and their corresponding comprehension questions are presented at a pace that is determined by the participant. The order of items is randomised. Participants then answer similarity ratings questions entitled by the corresponding 12 *SRT* items. Moreover, two sets of 15 scrambled sentences, taken from Savulich et al. [17], are counterbalanced across participants, so that half of participants received *SST* set 1 while the other half received *SST* set 2. Whether or not participants perform the *SRT* before the *SST,* or vice versa, is counterbalanced: half of participants perform the *SRT* first, while the other half performs the *SST* first, in order to minimise potential effects that one task might have on the performance on the other task
- Clinical symptoms assessed using the *PANSS*, *Paranoia Scale*, *Paranoid Thoughts Scale*, *PDI-21* and *HADS*, as well as the *Structured Clinical Interview for DSM-5* (*SCID-5*) [59] will be conducted by a trained Clinical Psychologist (AT) during baseline assessment

After receiving the intervention (CBM-pa or text-reading control) interim assessment (T_i1–5_) includes at each time point:

- Paranoid beliefs measured using a total of eight items of the *SRT*
- Paranoid symptoms assessed by *PANSS* item 6 (P6 Suspiciousness/Persecution) only

At week 6, after receiving the intervention, the assessment (T_1_) includes measures of:

- Paranoid beliefs (*SRT*, *SST* and *CFS*). As for T_0_, *SRT* and *SST* contain the same number of items (12 and 15, respectively) and are counterbalanced across participants
- Clinical symptoms (*PANSS* item 6, *Paranoia Scale*, *Paranoid Thoughts Scale*, *PDI-21* and *HADS*)
- Vulnerability to stress using the *Laughter Task* and *VRE* in counterbalanced order

Participants will be contacted at 1 and 3 months from end of the session 6 for follow-up assessments (T_2_–T_3_) which include:

- Paranoid beliefs measured, using a total of 12 items from the *SRT*
- Clinical symptoms (*PANSS* item 6, *Paranoia Scale*, *Paranoid Thoughts Scale*, *PDI-21* and *HADS*).

## Data monitoring

The researchers involved in the study will enter data into an SPSS spreadsheet. There will be four separate spreadsheets for each assessment phase (baseline, interim, end of intervention and follow-ups). The data will be stored on a locked group drive, only accessible to researchers in the ‘Emotion and Cognition’ group at IoPPN, King’s College London.

The separate SPSS spreadsheets will be merged, once all data have been entered and checked. The randomisation data and trial data will only be linked once all data collection is complete. All databases will be organised according to anonymised participant ID number (assigned at baseline) which will ensure that the databases are merged correctly.

## Risk management

Capacity to consent is assessed via a standard written protocol approved by the Ethics Committee [43]. The clinical psychologist will assess and ensure the safety of participants throughout the course of the study. A Risk Assessment Form has been developed and adapted from the protocol used at the Psychological Interventions Clinic for outpatients with Psychosis (PICuP) in order to minimise any potential adverse events. The study has been assessed as low risk, however, the *VRE* and *Laughter Task* could be perceived as mildly stressful for those experiencing paranoia. To address this, the nature of the task, including the option to withdraw, will be described in writing (Information Sheet) and reiterated verbally immediately prior to the relevant session. Mood state will be assessed before leaving and at follow-up contact. Any adverse events and serious adverse events will be recorded and reported in full in the trial report/journal paper. Any adverse events, defined as any events that result in death, serious injury or hospitalisation, which are considered by the chief investigator or Trial Management Group (TMG) to be related to trial procedures in any way, or are unexpected in that they are not listed in the trial protocol, will be reported promptly to the sponsor and the Ethics Committee.

## Analysis

### Statistical analysis

Analyses will be conducted using STATA 13 [60]. Descriptive statistics will be used to summarise clinical and demographic characteristics of patients. Feasibility of trial procedures will be examined using proportions and 95% confidence intervals for assessments of feasibility and acceptability in terms of recruitment, consent, dropout, follow-up and integrity of double blinding.

The variance observed in this sample will be used for sample size calculation for the future RCT. As recommended by Browne [37] and Lancaster and colleagues [39] the 80% upper one-sided confidence limit of the variance estimate rather than the variance estimate itself will be used. The final effect size for sample size calculations will be obtained by dividing the minimum clinically important difference by this variance estimate. We define a clinically important difference as a symptom score drop of ≥20%. To assess efficacy of the intervention using the *SRT* [41] a three-factorial within-and-between group design will be used with a random-effects model [61]. These analyses will follow the intention-to-treat principle, with data from all participants who entered the study included into the analysis. Emphasis will be on confidence interval of effect sizes estimation rather than hypothesis testing which allows us to assess the imprecision of the estimates. Rates and characteristics of dropout will be explored and where appropriate, statistical techniques for handling missing data will be used. Furthermore, to address a limitation given the size of the sample employed, statistical analyses will be corrected for multiple testing.

### Health economic analysis

As this is a feasibility study, a full economic evaluation is premature as the sample is small and costs are highly variable in this population which can lead to inaccurate results. However, piloting of economic outcomes and service use questionnaires is advisable. To assess the feasibility and acceptability of economic-related measures in this population, the Adult Service Use Schedule (*AD-SUS*) [62–64], European Quality of Life-5 Dimensions 3 level (*EQ-5D-3 L*) [63–65] and the 12-item Short Form Health Survey (*SF-12*) version 2 [66] will be piloted. We will not perform an economic evaluation but will explore completion rates and missing data for the *AD-SUS*, *EQ-5D-3 L* and *SF-12*. Additionally, we will explore resources described in the free-text sections of the *AD-SUS* to determine if any frequently used resources have been omitted. The *AD-SUS* will be used to measure individual-level resource use. It has been successfully applied in a range of adult mental health populations [62–64]. Further, for use in this study, the *AD-SUS* has been modified based on clinical expertise, and our LEAP service user group feedback. The *AD-SUS* records all-cause hospital- and community-based health- and social-care services including the use of psychotropic medication. The *AD-SUS* will be completed in interviews with participants and collected at baseline, at the end of intervention (6 weeks after randomisation) and at 3-month follow-up. The *EQ-5D-3 L* [65, 67, 68] is a self-completion instrument used to measure health-related quality of life. It consists of five domains (mobility, self-care, usual activities, pain/discomfort and anxiety/depression) and a rating of own health by means of a *VAS*. Respondents report difficulties in each area on three levels (none, some/moderate, extreme). The *SF-12* [66] is also a health-related quality of life measure. It consists of eight domains (physical functioning, social functioning, physical role limitations, emotional role limitations, energy, pain, mental health and general health perceptions). Two summary scores are produced: the mental health component score and the physical health component score. Both the *EQ-5D-3 L* and the *SF-12* will be completed at baseline and at 3-month follow-up. They can be used to generate health states, utility values and quality-adjusted life-years (QALYs) when appropriate.

### Qualitative analysis

Data will be transcribed by study researchers for thematic analysis. The LEAP group will be involved in identifying themes and codes, supported by McPin’s previous similar experience. Data will be analysed using the principles of grounded theory and framework analysis [69–71]. In this approach, categories or themes emerge naturally from the data during an iterative process of coding and revision that can identify recurrent patterns of views/experiences.

## Discussion

Persecutory delusions are at the severe end of a paranoia spectrum, associated with poorer health, emotional wellbeing and social functioning [2], and for which treatments need to be significantly improved. There is evidence that persecutory delusions are maintained by underlying cognitive biases which perpetuate distressing paranoid beliefs [72], specifically biases in the interpretation of emotional ambiguity [17, 18, 73]. Consequently, there are calls for new psychological treatments to be developed which target these maintaining mechanisms [72]. To date, there has been no intervention that uniquely targets the biased interpretation of emotional ambiguity that is known to be associated with clinical [18] and non-clinical [17] paranoia.

The current study will evaluate CBM-pa which directly targets biased interpretation of emotional ambiguity relevant to paranoia. If effective, CBM-pa could offer a new intervention to complement other psychological treatments (including CBT) [74]. New targeted interventions are required because CBT effect sizes for delusions are small to modest [75], a significant proportion of patients do not respond fully and resource limitations restrict availability [76]. This has been recognised by the advent of the IAPT-SMI (Increasing Access to Psychological Therapies for Severe Mental Illness) programme. The development of an evidence base for psychological treatments is seen as a priority for IAPT- SMI [77] and the evaluation of CBM-pa could contribute to these developments, as a potential low-cost, accessible and flexible intervention.

For more details about the trial, refer to the SPIRIT Checklist (Additional file 2).

## Trial status

Recruitment commenced in May 2016 and will continue until August 2017.

## Additional files

Additional file 1: Examples of CBM-pa and control conditions. **Figure S1a.** Instructions for the task are included within the programme and displayed before the participant begins. There is no time limit on the instructions. **Figure S1b.** A CBM-pa intervention passage. The passage is initially ambiguous, but the final word solution requires the participant to interpret in a non-paranoid way. Participants are initially given 20 s to read the passage, and 20 s to complete the word solution. **Figure S1c.** The participant is required to enter the first missing letter of the word, and is given positive feedback if they do so. **Figure S1d.** An incorrect response prompts the participant to try again, and more letters are given to help. Participants are given 23 s to respond. **Figure S1e.** The solution is then shown for 5 s regardless of whether a participant types the letter correctly or not. **Figure S1f.** A comprehension question is asked to encourage the participant to engage with the meaning of the passage. Participants are given 20 s to answer. **Figure S1g.** The figure above is shown when a participant responds in a non-paranoid way, for a maximum of 20 s. **Figure S1h** The figure above is shown when a participant responds in a paranoid way, for a maximum of 20 s. **Figure S1i.** A text-reading control passage. The text reading control programme is presented in an identical fashion to the CBM-pa. (DOCX 158 kb)

Additional file 2: SPIRIT 2013 Checklist: recommended items to address in a clinical trial protocol and related documents. (DOC 120 kb)

## Abbreviations

CBM: Cognitive Bias Modification
CBT: Cognitive behavioural therapy
LEAP: Lived Experience Advisory Panel
MRC: Medical Research Council
NHS: National Health Service
NICE: National Institute for Health and Care Excellence
RCT: Randomised controlled trial
TAU: Treatment as Usual
TSG: Trial Steering Group
