# Supplementary material for: Cognitive Bias Modification for paranoia (CBM-pa): study protocol for a randomised controlled trial
Source: Trials. 2017 Jun 29;18:298. doi: 10.1186/s13063-017-2037-x (PMC5492504; doi:10.1186/s13063-017-2037-x)
Supplement: Supplementary file 1 — Examples of CBM-pa and control conditions. Figure S1a. Instructions for the task are included within the programme and displayed before the participant begins. There is no time limit on the instructions. Figure S1b. A CBM-pa intervention passage. The passage is initially ambiguous, but the final word solution requires the participant to interpret in a non-paranoid way. Participants are initially given 20 s to read the passage, and 20 s to complete the word solution. Figure S1c. The participant is required to enter the first missing letter of the word, and is given positive feedback if they do so. Figure S1d. An incorrect response prompts the participant to try again, and more letters are given to help. Participants are given 23 s to respond. Figure S1e. The solution is then shown for 5 s regardless of whether a participant types the letter correctly or not. Figure S1f. A comprehension question is asked to encourage the participant to engage with the meaning of the passage. Participants are given 20 s to answer. Figure S1g. The figure above is shown when a participant responds in a non-paranoid way, for a maximum of 20 s. Figure S1h The figure above is shown when a participant responds in a paranoid way, for a maximum of 20 s. Figure S1i. A text-reading control passage. The text reading control programme is presented in an identical fashion to the CBM-pa. (DOCX 158 kb) [file 13063_2017_2037_MOESM1_ESM.docx]

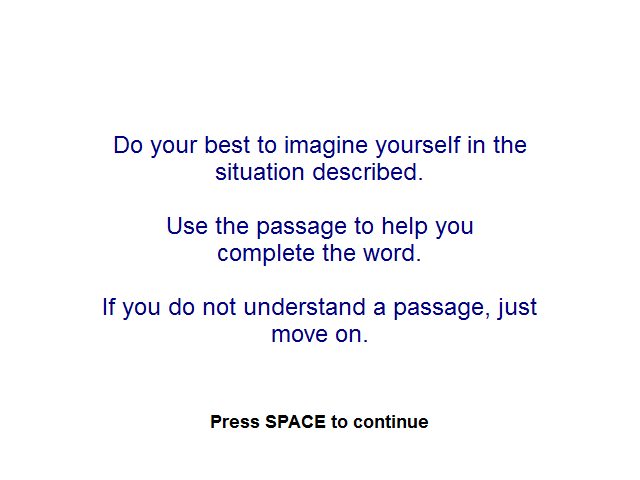


**Figure S1a.** Instructions for the task are included within the program and displayed before the participant begins. There is no time limit on the instructions.


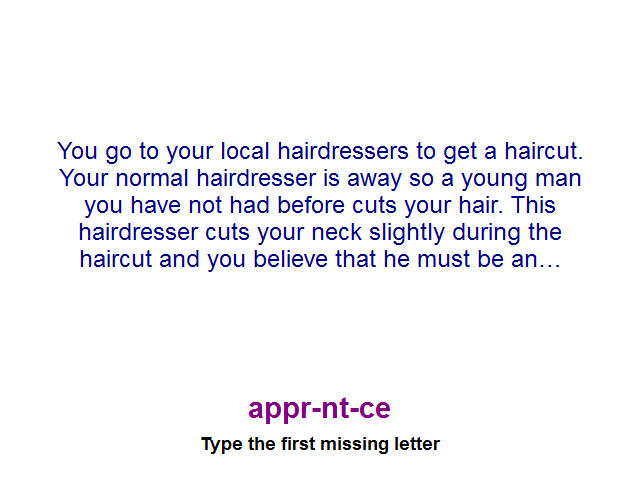


**Figure S1b.** A CBM-pa intervention passage. The passage is initially ambiguous, but the final word solution requires the participant to interpret in a non-paranoid way. Participants are initially given 20 seconds to read the passage, and 20 seconds to complete the word solution.


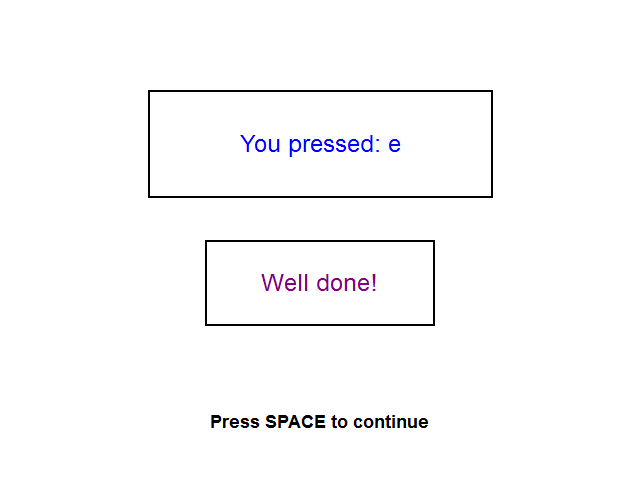


**Figure S1c.** The participant is required to enter the first missing letter of the word, and is given positive feedback if they do so.


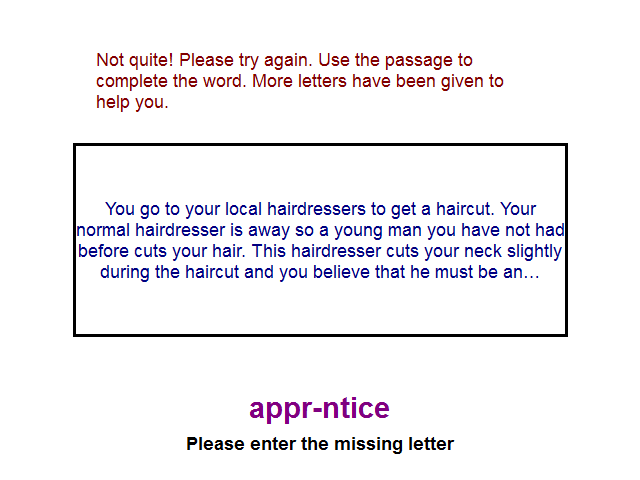


**Figure S1d.** An incorrect response prompts the participant to try again, and more letters are given to help. Participants are given 23 seconds to respond.


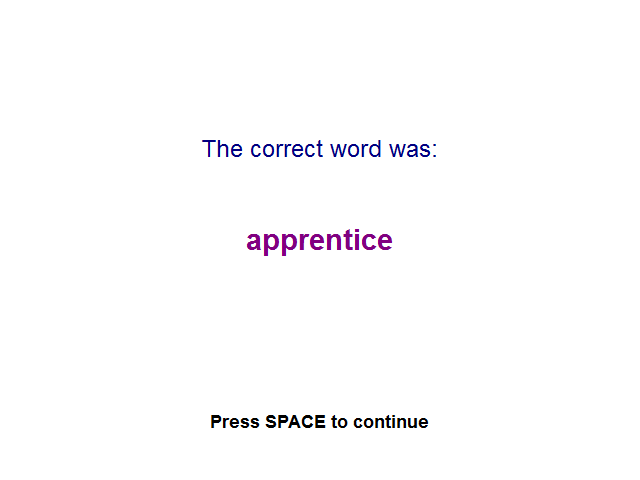


**Figure S1e.** The solution is then shown for 5 seconds regardless of whether a participant types the letter correctly or not.


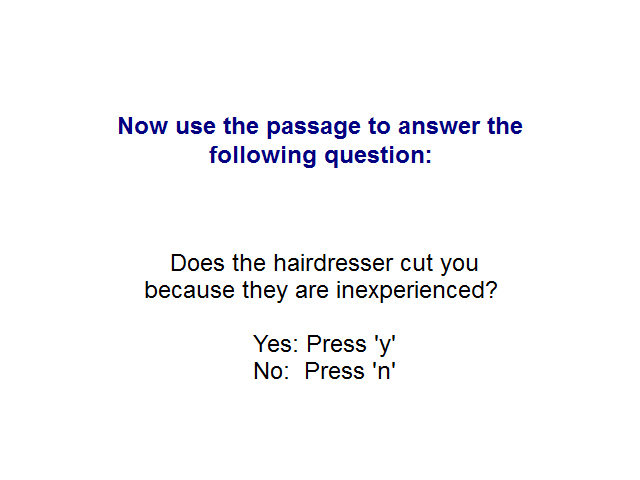


**Figure S1f.** A comprehension question is asked to encourage the participant to engage with the meaning of the passage. Participants are given 20 seconds to answer.


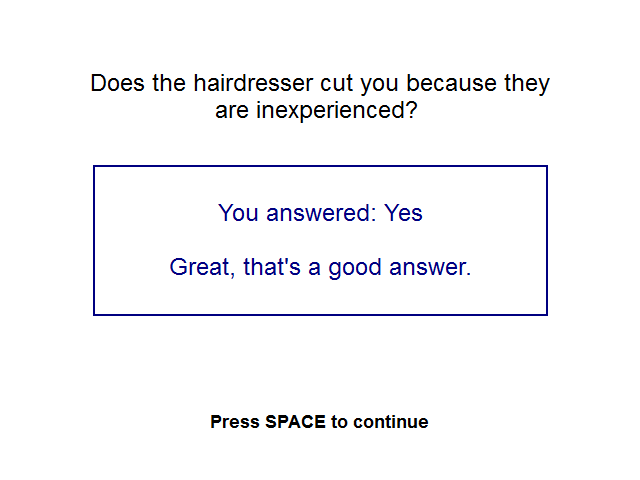


**Figure S1g.** The figure above is shown when a participant responds in a non-paranoid way, for a maximum of 20 seconds.


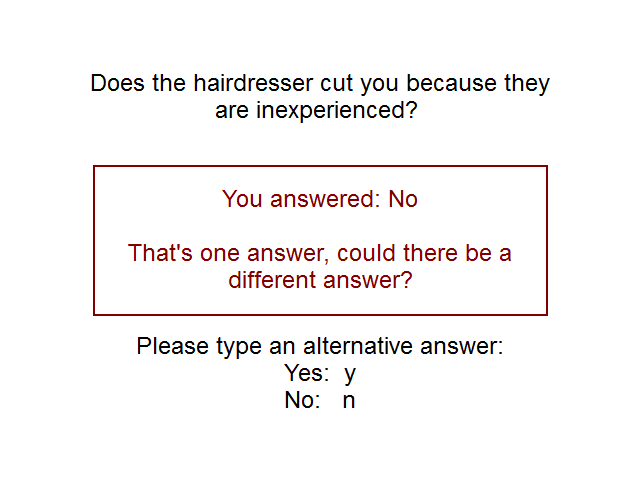


**Figure S1h.** The figure above is shown when a participant responds in a paranoid way, for a maximum of 20 seconds.


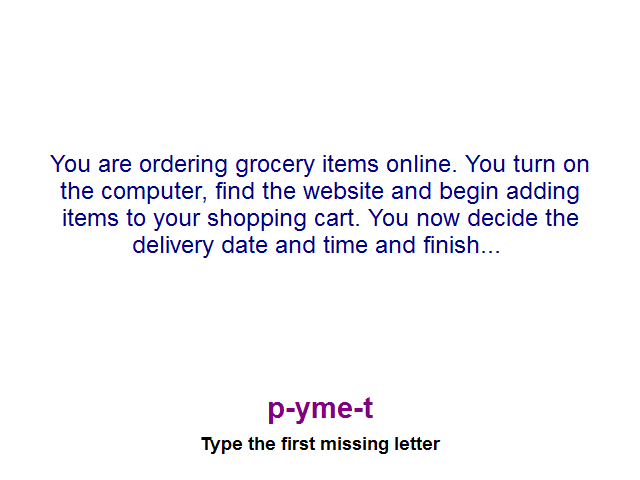


**Figure S1i**. A text-reading control passage. The text reading control programme is presented in an identical fashion to the CBM-pa.
